# Buffered Lugol's Iodine Preserves DNA Fragment Lengths

**DOI:** 10.1093/iob/obae017

**Published:** 2024-06-14

**Authors:** P M Gignac, D Valdez, A C Morhardt, L M Lynch

**Affiliations:** Department of Cellular and Molecular Medicine, University of Arizona, Tucson, AZ 85724, USA; Department of Anatomy, Midwestern University, Glendale, AZ 85308, USA; Department of Neuroscience, Washington University in St. Louis, St. Louis, MO 63130, USA; Department of Anatomy, Midwestern University, Glendale, AZ 85308, USA

## Abstract

Museum collections play a pivotal role in the advancement of biological science by preserving phenotypic and genotypic history and variation. Recently, contrast-enhanced X-ray computed tomography (CT) has aided these advances by allowing improved visualization of internal soft tissues. However, vouchered specimens could be at risk if staining techniques are destructive. For instance, the pH of unbuffered Lugol's iodine (I_2_KI) may be low enough to damage deoxyribonucleic acid (DNA). The extent of this risk is unknown due to a lack of rigorous evaluation of DNA quality between control and experimental samples. Here, we used formalin-fixed mice to document DNA concentrations and fragment lengths in nonstained, ethanol-preserved controls and 3 iodine-based staining preparations: (1) 1.25% weight-by-volume (wt/vol.) alcoholic iodine (I_2_E); (2) 3.75% wt/vol. I_2_KI; and (3) 3.75% wt/vol. buffered I_2_KI. We tested a null hypothesis of no significant difference in DNA concentrations and fragment lengths between control and treatment samples. We found that DNA concentration decreases because of staining—potentially an effect of measuring intact double-stranded DNA only. Fragment lengths, however, were significantly higher for buffered I_2_KI and control samples, which were not, themselves, significantly different. Our results implicate buffered I_2_KI as the appropriate choice for contrast-enhanced CT imaging of museum wet collections to safely maximize their potential for understanding genetic and phenotypic diversity.

## Resumen

Las colecciones de museos juegan un papel crucial en el avance de la ciencia biológica al preservar la historia y la variación fenotípica y genotípica. Recientemente, la tomografía computarizada (CT) mejorada con contraste ha facilitado estos avances al permitir una mejor visualización de los tejidos blandos internos. Sin embargo, los especímenes con vales podrían estar en riesgo si las técnicas de tinción son destructivas. Por ejemplo, el pH del yodo de Lugol sin tamponar (I_2_KI) puede ser lo suficientemente bajo como para dañar el ADN. Se desconoce el alcance de este riesgo debido a la falta de una evaluación rigurosa de la calidad del ADN entre las muestras de control y las experimentales. Aquí utilizamos ratones fijados en formalina para documentar las concentraciones de ADN y las longitudes de los fragmentos en controles no teñidos, preservados en etanol, y en tres preparaciones de tinción basadas en yodo: (i) 1.25% peso/volumen (wt/vol.) de yodo alcohólico (I_2_E), (ii) 3.75% wt/vol. I_2_KI, y (iii) 3.75% wt/vol. I_2_KI tamponado. Probamos una hipótesis nula de que no hay diferencias significativas en las concentraciones de ADN y las longitudes de los fragmentos entre las muestras de control y las de tratamiento. Encontramos que la concentración de ADN disminuye debido a la tinción, potencialmente un efecto de medir solo ADN de doble cadena intacto. Sin embargo, las longitudes de los fragmentos fueron significativamente mayores para I_2_KI tamponado y las muestras de control, que no fueron, ellas mismas, significativamente diferentes. Nuestros resultados implican que I_2_KI tamponado es la opción adecuada para la imagenología CT mejorada con contraste de colecciones húmedas de museos para maximizar de manera segura su potencial para comprender la diversidad genética y fenotípica.

## Introduction

Nano- and microscale X-ray computed tomography (nCT and µCT, respectively) have fundamentally changed the trajectory of morphological research by allowing nondestructive, internal visualization and digital sharing of phenotypic complexity. Standard CT scanning enables the three-dimensional (3D) imaging and quantification of the densest animal tissues—bones and teeth—from living and fossil organisms. As a tool for the anatomical sciences, CT imaging has promoted new anatomical discoveries by facilitating the characterization of internal traits for phylogenetic analysis, the modeling of biomechanical function, the study of ecological variation, and the retracing of evolutionary innovations (e.g., Rae et al. 2002; Ross 2005; Witmer et al 2008; Brainerd et al. 2010; Hilton et al. 2021). Historically, these insights were limited to naturally radiopaque features because those containing predominantly water (i.e., soft tissues) cannot meaningfully attenuate X-rays, which is necessary for CT imaging (Ketcham and Carlson 2001). The recent addition to CT scanning of contrast-enhancing agents such as iodine, osmium tetroxide, and phosphomolybdic acid, among others, was an important advancement in the field (Baird and Taylor 2017). These chemical stains render soft-tissue features radiopaque, and the results appear like those of magnetic resonance imaging—enabling differentiation of low-density structures but at far higher spatial resolutions (Gignac et al. 2016; Gignac and Kley 2018). This insight has substantially added to the richness, detail, and fidelity of digital morphological data that are now gleaned using X-ray imaging modalities.

As a result of its broad utility, contrast-enhanced nCT and µCT imaging have become commonplace in morphology-focused research labs. One of the most routinely deployed techniques is diffusible iodine-based contrast-enhanced CT (diceCT; Gignac et al. 2016; Heimel et al. 2019; Callahan et al. 2021; Gray et al. 2023; Kolmann et al. 2023). Pioneering work by Metscher (2009a, 2009b) on vertebrate embryonic samples showed that Lugol's iodine (iodine potassium iodide, I_2_KI) is an effective agent for soft-tissue contrast enhancement in CT imaging. Expanding and building on Metscher's protocols has yielded spectacular imagery of nerves, muscles, glands, special sensory structures, blood vessels, and epithelia in a diverse array of invertebrates, vertebrate embryos, and larger, postembryonic vertebrates. Because of its ease of use, reliable staining, reduced toxicity, low cost, and visual reversibility (Gignac et al. 2016), researchers often choose diceCT over other contrasting agents. This has enabled research teams to visualize, study, and share the delicate, developmental, functional, and phylogenetic traits of their model organisms with colleagues all over the world (Blackburn et al. 2024). Altogether, this has increased the pace of scientific discovery by enabling previously unaddressable questions to be answered (Orsbon et al. 2018; Yohe et al. 2018; Rawson et al. 2020).

Iodine-stained samples are usually chemically fixed prior to staining to preserve their physical integrity over time (Gignac et al. 2016). Fixatives, such as formalin, not only promote the preservation of morphology but also create significant challenges for sampling deoxyribonucleic acid (DNA) (Vachot and Monerot 1996; Tang 2006; Speer et al. 2022). This is due to its effect of cross-linking biomolecules, which causes fragmentation that complicates DNA extraction (Hamazaki et al. 1993; Koshiba 1993; Hoffman et al. 2015). Early efforts to extract usable DNA from such specimens often resulted in low yields and degraded DNA (Schander and Kenneth 2003), limiting the genomic utility of vouchered samples stored in collections worldwide. However, the development of techniques over the last three decades that are designed for ancient and archival DNA (aDNA) extraction has greatly expanded the sampling of historical genomes (Taubenberger et al. 1997; Coombs et al. 1999; Reid et al. 1999). By adopting protocols that mitigate formalin-induced alteration, such as the use of heat and alkali treatments to break protein–DNA cross-linkages caused by formalin exposure (Hykin et al. 2015; Brino et al. 2023), researchers now routinely recover genomic information from specimens that were once considered too degraded or altered for genetic analysis (Zimmermann et al. 2008; Jaksch et al. 2016; Straube et al. 2021). This progress has not only facilitated the preservation of DNA integrity in the face of potential damage but also opened new avenues for the retrospective study of biodiversity, evolution, and conservation genetics through DNA bar coding of museum and archival specimens (e.g., Scherz et al. 2020).

Retrospective research is only possible because museum collections play a pivotal role in preserving and recording natural history and its variation. They offer a critical resource for studying morphological and molecular evolution. Together, diceCT and aDNA techniques provide the potential to study how these aspects of biodiversity correlate by drawing distinct data from the same samples. One limit to this potential is that preserved specimens can be exposed to numerous degrading chemical environments due to fixation, storage, and contrast enhancement. (Note that neither ethanol storage nor ionizing radiation from CT scanning adversely affects DNA integrity [Stein et al. 2013; Hall et al. 2015].) Often, collections-access policies are crafted to maximize potential uses of their specimens (e.g., scientific, educational, archival; Simmons 2014), and curators and collections managers are tasked with evaluating whether or not to permit potentially destructive access that may preclude future sampling of a specimen for additional research (Clemann et al. 2014). For example, exposing specimens to low-pH environments (≤4.5 pH), which can result from the mixing and especially long-term storage of staining agents (Gottardi 2015), may cause organ-, tissue-, and cellular-level distortions (Simmons 2014). Highly acidic environments deform and shrink whole specimens by altering osmotic pressure, which withdraws water from a sample (Dawood et al. 2021), or lead to demineralization by dissolving calcium deposits in osseous structures (Zimmerman et al. 2008; Sano et al. 2022). Additionally, highly acidic compounds disrupt DNA integrity by causing depurination/depyrimidination (Zimmerman et al. 2008). Addressing these problems typically involves the use of phosphate-based buffering agents (Zimmerman et al. 2008; Gottardi 2015; Early et al. 2020; Dawood et al. 2021), with generally acceptable levels of success that are relatively easy to determine through visual inspection of undistorted gross morphologies or CT scanning of a specimen's skeletal features. The exception is for metrics of DNA integrity (e.g., concentration, fragment length, and sequence reproducibility), which require deliberate study and comparison to control samples. As a result, the potential loss of DNA integrity from iodine staining of fixed specimens remains an unaddressed concern for those sampling preserved specimens (Hall et al. 2015; Hsu et al. 2016).

To date, there have been no rigorous evaluations of differences in DNA metrics between control and contrast-enhanced experimental samples, like those undergoing diceCT protocols. The formation of triiodide (I_3_^−^; the form iodine predominantly takes in solution) and other iodine species has the potential to create an acidic environment (Gottardi 2015; Kolmann et al. 2023)—highly acidic in the case of relatively strong iodine concentrations (Gottardi 2015; Early et al. 2020; Dawood et al. 2021). Such environments are known to negatively impact sample integrity, including DNA quality (Zimmerman et al. 2008). Fortuitously, an updated form of Lugol's iodine has recently been proposed: buffered Lugol's (B-Lugol; *sensu*  Dawood et al. 2021), which incorporates a Sørensen's phosphate buffer into the I_2_KI contrasting solution. The buffer maintains a neutral pH (∼7.2) for the duration of staining, regardless of I_3_^−^ concentration, and was developed specifically to mitigate tissue distortions. However, B-Lugol may also be critical for DNA integrity because of its ability to reduce or eliminate DNA damage associated with high acidity. If this is the case, B-Lugol will enable diceCT imaging of vouchered specimens without the issue of DNA fractionation. Here, we address this potential benefit by evaluating the magnitude of differences in DNA quality using formalin-fixed mouse samples under four preparations: (1) ethanol only (nonstained as a control); (2) ethanol-based iodine stain; (3) unbuffered Lugol's iodine; and (4) buffered Lugol's iodine. We collected DNA concentration and fragment length measurements to test the null hypothesis that DNA quantity and quality between unstained specimens and those stained with unbuffered (e.g., ethanol-based iodine stain is included in this category) and buffered iodine solutions do not differ significantly. We predict, however, that nonstained samples and those subjected to buffered Lugol's protocols will provide greater DNA concentrations and yield longer fragment sizes relative to those prepared with the unbuffered iodine stain. We found that DNA concentration decreases because of staining, but fragment lengths were significantly higher for buffered I_2_KI and control samples. Importantly, buffered I_2_KI and control samples were not, themselves, significantly different. We conclude by making recommendations to help ensure museum collections remain multifunctional and continue to maximize their potential uses.

## Methods

### Tissue extraction

Twenty-five mice (*n* = 11 female, *n* = 14 male *Mus musculus*; Wards Science, Rochester, NY, USA), preserved in 10% formalin, were sampled for this study. Preservation was consistent across all specimens. Hearts, livers, and skin sections were extracted because they are common tissues obtained from museum specimens for genetic analysis (McDonough 2018; Tsai et al. 2020; Straube et al. 2021; Hahn et al. 2022). One skin patch (excluding underlying fascial layers) for each specimen was extracted from the abdominal wall. Intact livers and hearts (excluding pericardia) were also completely removed. All samples were manually extracted, and to preclude genetic contamination, the organ material for each specimen was retained in separate containers and handled with nitrile gloves and sterilized tools.

### Chemical preparation

Extracted tissues were dehydrated (i.e., an ethanol step-up protocol) using a series of ethanol baths to mimic typical vouchered museum specimen preservation conditions (Simmons 2014). The dehydration procedure first included a 25% ethanol bath for 24 h, followed by 40% and 55% ethanol baths each for an additional 24 h, and was completed in 70% ethanol for 72 h before staining. Mass reduction of biological tissues during ethanol dehydration is common (Simmons 2014; Hedrick et al. 2018); therefore, all samples were weighed at each stage to document shrinkage (Supplementary Table S1).

Following dehydration, each sample of heart, liver, and skin was quartered into equal-weight subsamples (Supplementary Table S1). One quarter was retained as a nonstained control, exposed only to the dehydration ethanol step-up protocol, and stored in 70% ethanol, while other samples were stained. The remaining three quarters were each used for a distinct staining protocol common for diceCT imaging: (1) 1.25% weight-by-volume (wt/vol.) I_2_E; (2) 3.75% wt/vol. I_2_KI (in distilled water); or (3) 3.75% buffered I_2_KI (in distilled water). (Note that I_2_ crystals are item AA4195536 from ThermoFisher Scientific Waltham, MA; KI crystals are item 746428-50G; powdered KH_2_PO_4_ is item NC0229895; powdered NA_2_HPO_4_ is item NC0229893—each from Sigma–Aldrich Burlington, MA.) All samples were fully submerged in their respective staining solution until completely stained (∼24–48 h, depending on stain concentration and specimen size; Dawood et al. 2021). Staining completeness was evaluated by visual confirmation that the tissue was uniformly dark both externally and internally at the centermost point. The pH of 3.75% I_2_KI and 3.75% buffered I_2_KI solutions was measured using an optical pH reader (Fisherbrand, Pittsburgh, PA, pH/ATC tester no. 3057763) at the time of mixing and after 48 h to document differences in the staining environment. Once staining was complete, all samples were then destained by leaching iodine from each tissue, using 70% ethanol (Gignac et al. 2016; Early et al. 2020). Destaining baths were refreshed daily until visible leaching ceased (∼5–6 days, depending on the amount of iodine absorbed and specimen size). The 70% ethanol control was not changed. As a result of this protocol, noncontrol samples passed through the following series of chemical preparations: formalin fixation, ethanol dehydration, iodine staining, and ethanol-based destaining. Control samples passed through steps of formalin fixation, ethanol dehydration, and ethanol storage. Because they were meant to simulate fixed and ethanol-stored museum samples, controls did not undergo additional steps unrelated to wet storage. In order to mimic typical genetic extraction protocols from 70% ethanol-stored museum specimens, we did not conduct a rehydration (i.e., an ethanol step-down) protocol prior to extracting DNA. (Note that various chemical washes are routine for aDNA protocols [e.g., Coombs et al. 1999; Straube et al. 2021] in order to optimize the environment for DNA extraction; we compare our control results with those from the aDNA literature [see the “Discussion” section] to ensure that our baseline DNA signal matches what would be expected from previous studies.)

### Extraction protocol

Standardized tissue extraction followed the protocol described in Lynch (2019). Approximately 25 mg of tissue was removed from each destained sample and subjected to a proteinase-K (8.75 μL 20 mg/mL), RNase A (5 μL 1 mg/mL), dithiothreitol (DTT) (10 μL 5 mM), and lysis buffer digestion (125 μL stock of 50 mM Tris–HCl pH 8, 20 mM ethylenediaminetetraacetic acid [EDTA] pH 8, 2% sodium dodecyl sulfate) (item nos. AM254612091012, AAJ1539703, Trizma base T1503-100G, E7889-100ML, and 1555307, respectively; purchased from Fisher Scientific International Inc., Pittsburgh, PA, USA, and Sigma–Aldrich), followed by a phenol:chloroform:isoamyl (25:24:1) extraction protocol (Bello et al. 2001; Lynch 2019). We chose this protocol because it has been shown to be the most effective extraction method for museum formalin-fixed wet specimens (McDonough 2018; Tsai et al. 2020; Straube et al. 2021; Hahn et al. 2022). Tissue samples in solution were broken down by heating at 55°C for 72 h in a Fisher Scientific Isotemp oven (Fisher Scientific International Inc.). This period is longer than the standard requirement of 24 h for thermal tissue breakdown (Lynch 2019), but it was necessary because our samples were chemically preserved, requiring additional thermal exposure. We eluted all samples in 80 μL of elution buffer (1× TE buffer). (Note that samples from one specimen, M32, were damaged during a centrifugation step, resulting in the loss of all four of its heart samples, buffered I_2_KI liver and skin samples, as well as control and I_2_E liver samples.)

### DNA analyses

We analyzed DNA concentration for each sample extraction using a Qubit 4 Fluorometer (ThermoFisher Scientific, Waltham, MA, USA) and a double-stranded (ds) DNA Broad Range Assay Kit (ThermoFisher Scientific). DNA fragment size was measured for each extraction using an Agilent 5200 Fragment Analyzer System (Agilent, Santa Clara, CA, USA) and an HS NGS Fragment Kit (1–6000 bp) at the Idaho State University Molecular Research Core Facility (RRID:SCR_012598).

### Statistical analyses

We evaluated differences in DNA concentration and DNA fragment size using two-way analyses of variance, followed by Tukey’s post hoc tests to evaluate treatment groups (*P* < 0.05). For both DNA concentration and fragment size, we tested for differences within each staining protocol while controlling for tissue type. We quantified the representative fragment size of each sample as the fragment length (bp) with the highest concentration (ng/μL) of dsDNA (i.e., the highest peak fragment size within each sample's distribution curve). All analyses were run in PAST 4.03 (Hammer et al. 2001).

## Results

**Fig. 1:**
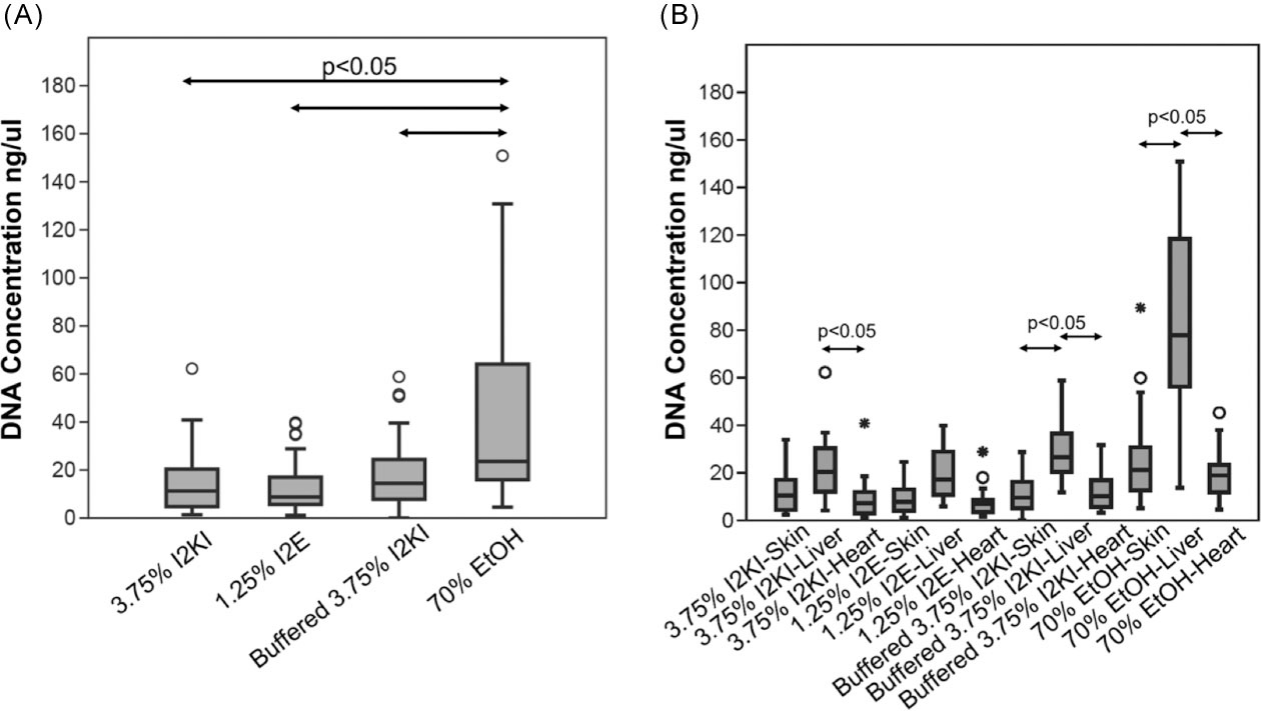
Box and whisker plot of DNA concentration by (**A**) protocol and (**B**) protocol and tissue type. Circles and asterisks indicate outlier samples outside the third quartile. Arrows indicate pairings whose DNA concentrations significantly differ (*P* < 0.05).

We examined differences in dsDNA concentrations across multiple comparisons. Contrasting the results for staining protocols (Fig. 1A), we found that dsDNA concentration was (1) significantly reduced in all specimens subjected to one of the three staining protocols as compared to the ethanol control samples (all *P*-values <0.001; Table 1) and (2) none of the staining protocols differed significantly from each other (see *P*-values listed in Table 1). Contrasting results for tissue types (Fig. 1B), we found that dsDNA concentration was (1) not significantly different across tissue types stained with I_2_E (see *P*-values listed in Table 2); however, (2) within the buffered Lugol's and control protocols, significant differences were found between skin and liver (all *P*-values <0.01; Table 2) and between (3) heart and liver (all *P*-values <0.05; Table 2). In these cases, liver samples show two to four times higher levels of dsDNA concentration. Raw values can be found in Supplementary Table S2.

**Table 1:** *P*-values for Tukey’s pairwise comparisons of DNA concentration between stain protocols

Stain protocol	I_2_KI	I_2_E	Buffered	EtOH
I_2_KI		0.8225	0.6214	**2.35E-13**
I_2_			0.1634	**2.16E-13**
Buffered				**2.16E-13**

Significantly different values indicated in bold.

**Table 2:** *P*-values for Tukey’s pairwise comparisons of DNA concentration within protocols between tissue types

Tissue	I_2_KI	I_2_E	Buffered	EtOH
Skin–liver	0.2613	0.142	**0.001469**	**2.14E-13**
Skin–heart	0.9902	1	1	0.8061
Liver–heart	**0.03443**	0.06308	**0.001951**	**2.14E-13**

Significantly different values indicated in bold.

**Fig. 2:**
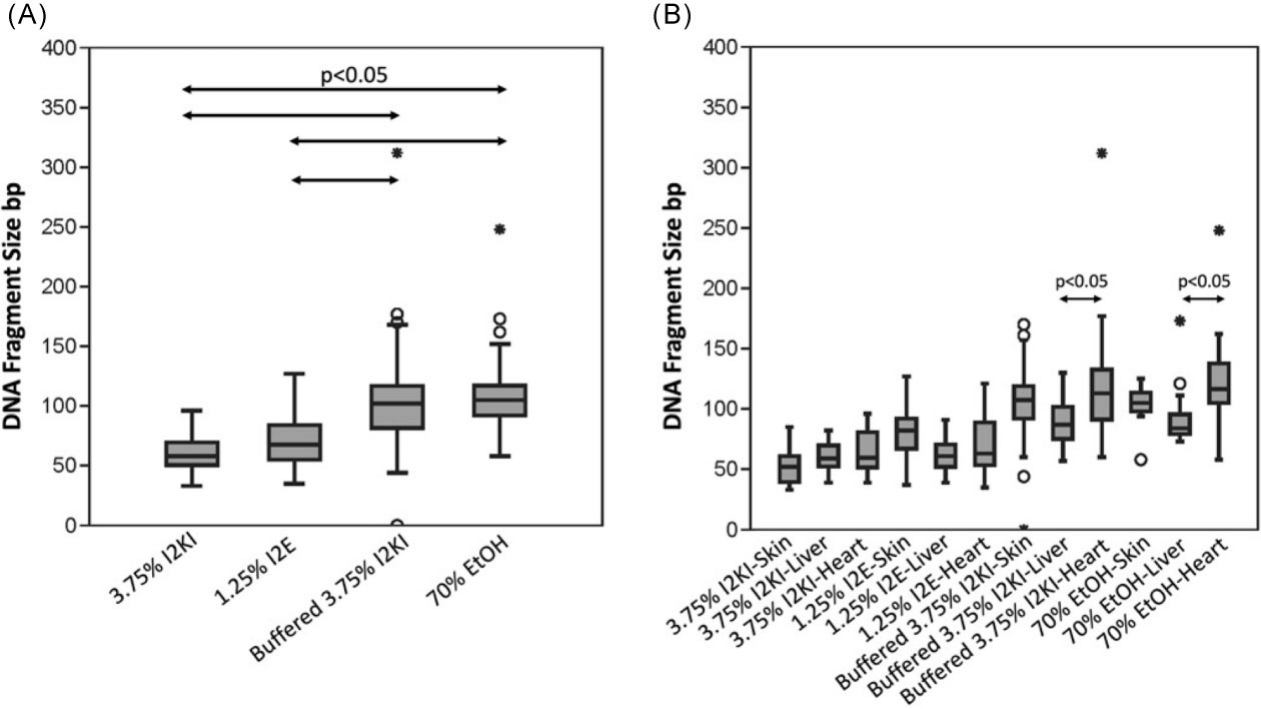
Box and whisker plot of DNA peak fragment size by (**A**) protocol and (**B**) protocol and tissue type. Circles and asterisks indicate outlier samples outside the third quartile. Arrows indicate pairings whose DNA peak fragment sizes significantly differ (*P* < 0.05).

We also examined differences in dsDNA fragment sizes across multiple comparisons. Contrasting the results for staining protocols (Fig. 2A), we found that (1) buffered 3.75% Lugol's iodine retained significantly larger fragments of dsDNA during the staining process than samples subjected to 1.25% wt/vol. I_2_E (*P* < 0.001; Table 3) and 3.75% wt/vol. I_2_KI (*P* < 0.001; Table 3) and (2) specimens stained in buffered 3.75% Lugol's iodine retained dsDNA fragment sizes that do not significantly differ from control samples (*P* = 0.9386; Table 3). Contrasting results for tissue types (Fig. 2B), dsDNA fragment size differed significantly within (1) buffered 3.75% Lugol's and (2) control samples. Specifically, heart samples retained larger fragments than liver samples in both preparations (*P*  < 0.001; Table 4), but neither heart and skin samples nor skin and liver samples significantly differed from one another (see *P*-values listed in Table 4). Raw values can be found in Supplementary Table S2.

**Table 3:** *P*-values for Tukey’s pairwise comparisons of DNA fragment size between stain protocols

	I_2_KI	I_2_E	Buffered	EtOH
I_2_KI		**0.0362**	**2.47E-13**	**2.31E-13**
I_2_			**5.85E-13**	**2.58E-13**
Buffered				0.9386

Significantly different values indicated in bold.

**Table 4:** *P*-values for Tukey’s pairwise comparisons of DNA fragment size within protocols between tissue types

Tissue	I_2_KI	I_2_E	Buffered	EtOH
Skin–liver	0.9551	0.2649	0.4581	0.6145
Skin–heart	0.6701	0.9287	0.3994	0.169
Liver–heart	0.9987	0.9503	**0.001064**	**0.000521**

Significantly different values indicated in bold.

Values of pH for 3.75% I_2_KI started at 6.9 and dipped to 4.6 after 48 h, whereas values for 3.75% buffered I_2_KI started at 7.5 and changed to 7.1 after 48 h. These values are consistent with those reported by Early et al. (2020) and Dawood et al. (2021) for similar mixtures of staining solutions.

## Discussion

In this study, we examined DNA concentrations and fragment lengths for soft-tissue samples prepared under a variety of iodine-staining conditions, reflective of the diceCT protocol, against controls. We found that our null hypothesis (dsDNA quantity and quality between unstained specimens and those stained with unbuffered and buffered iodine solutions do not differ significantly) was not supported. This was reflected by the lack of support for our prediction that buffered Lugol's protocols yield greater DNA concentrations relative to unbuffered protocols. However, we did find support for our prediction of longer fragment sizes for samples stained using buffered iodine relative to those prepared with the unbuffered iodine stain. Here, we discuss our findings in the context of other studies of DNA integrity, why results may have differed between the organs sampled, and how iodine exposure impacts DNA quality.

### DNA integrity

The dsDNA concentrations and fragment lengths obtained in our control (i.e., formalin-fixed, ethanol-stored) specimens are consistent with previous findings from preserved soft-tissue samples. Fixatives, such as formalin and formaldehyde, cause DNA denaturation, DNA-to-protein cross-linkage, and nucleic acid methylation (Hamazaki et al. 1993; Koshiba 1993; Hoffman et al. 2015). As a result, specimens treated with these fixatives consistently return lower yields of DNA with smaller fragment sizes (e.g., Hamazaki et al. 1993; Ferrer 2007; Katarina 2018). Studies focused on improving DNA extraction protocols in museum specimens (formalin or formaldehyde preserved and ethanol stored) consistently returned low quantities of highly fragmented DNA using the same or comparable extraction protocols to those used in our study (McDonough 2018; Tsai et al. 2020; Straube et al. 2021; Hahn et al. 2022). For example, DNA concentrations ranged from 33 to 2550 ng (Hahn et al. 2022), 17 to 937 ng/mg (Tsai et al. 2020), and 0.1 to 43 ng/μL (McDonough 2018), and fragment sizes averaged between 25 and 200 bp in length (Hamazaki et al. 1993; Koshiba 1993; Hoffman et al. 2015). This compares to an average concentration of 3316 ng (133 ng/mg or 41 ng/μL) and an average fragment length of ∼100 bp in our control samples. The results indicate that (1) our formalin-fixed, ethanol-preserved control samples accurately represent dsDNA quality found in most museum wet specimens and (2) our extraction protocols successfully returned dsDNA concentrations and fragment sizes predicted by previous studies for these types of tissues. Further comparisons of dsDNA quality from iodine-stained samples to our control are, therefore, appropriate and reflect damage induced by the addition of iodine.

### Organ-specific differences

We sampled tissues from three organs: heart, skin, and liver. We found systematic differences in DNA fragment lengths across our three sample types, regardless of iodine preparation. Although high levels of variance in fragment length caused skin samples not to differ significantly when compared to each of heart and liver, we generally found that average fragment lengths were longest for cardiac samples, intermediate for skin samples, and shortest for liver samples. We hypothesize that this reflects the mechanical complexity of each of the tissues sampled. Formalin fixation cross-links proteins already present in tissues (Simmons 2014), and this process toughens otherwise compliant samples. With an abundance of actin and myosin proteins, cardiac muscle stands to be reinforced the most when fixed with formalin (see Addis et al. 2009). On the other hand, liver tissue is a composite of parenchymal and nonparenchymal cells with a comparably low volume of structural proteins (Koui et al. 2017). As a result, the physical breakdown of samples through mechanical and thermal degradation is likely to have the greatest impact on liver samples, followed by skin, and heart. dsDNA strands in less reinforced samples, like those of the liver, are therefore also more exposed to breakdown during the extraction phase of our protocol. This is in stark contrast to fixed cardiac tissues, whose cytoarchitectural structure resembles an entangled protein matrix (Addis et al. 2009), capable of physically protecting dsDNA and, presumably, limiting its fragmentation.

### Impacts of iodine exposure

We exposed our organ samples to three staining protocols, and we observed significant reductions in DNA fragment lengths and concentrations based on exposure to different iodine preparations. Regarding fragmentation lengths, we found that unbuffered iodine solutions (1.25% I_2_E, 3.75% I_2_KI) showed lower fragment lengths than buffered and controls. Moreover, fragment lengths in buffered and control samples were not significantly different. We interpret that the high acidity (<5 pH; also see Dawood et al. 2021) of unbuffered iodine solutions fragments dsDNA strands, resulting in systematically shorter fragments available to be read in 1.25% I_2_E- and 3.75% I_2_KI-prepared samples. We hypothesize that this effect may be due to the malfunction of histones in highly acidic environments. For example, aspartic acid and histidine normally harbor a deprotonated carboxyl group (Voet et al. 2016). These histones can catalyze DNA cleavage in nonphysiological, low-pH environments when protonated (Ren et al. 2022). Our observations, therefore, support the notion that keeping pH neutral during staining ensures that dsDNA structure is retained. Importantly, we measured no degradation in dsDNA fragment length in buffered Lugol's iodine as compared to our control sample, indicating that buffered I_2_KI does not risk excessive fragmentation of dsDNA.

We found that in all cases (1.25% I_2_E, 3.75% I_2_KI, and 3.75% B-I_2_KI), iodine staining reduces dsDNA concentrations and that these concentrations were similar across all three iodine preparations (i.e., as compared to an organ sample). We do not interpret this difference to reflect bias in our methodology of concentration measurement. We established dsDNA concentrations using fluorescent dye tagging (Seshadri 2019). Qubit dyes have a positive charge, enabling them to bind to phosphate groups in dsDNA, which are negatively charged. The dyes bind to specific locations (e.g., the major groove) along the double helix, but they do not disrupt the structure of dsDNA. If triiodide altered the Qubit methodology, we would have expected the positively charged dye to interact with negatively charged iodine species (e.g., I_3_^−^). This would have been indicated by falsely high readings of DNA concentration, which we did not observe. Additional considerations include our use of phenol:chloroform:isoamyl extraction to separate dsDNA from lipids and other cellular debris, EDTA to sequester positively charged metal ions, sodium acetate to neutralize charges on the DNA backbone, and DTT to release DNA from histones and other protective proteins. However, negatively charged iodine species do not seem to react with these chemicals either, perhaps with the exception that triiodide will dissolve in chloroform (both are nonpolar molecules; Hildebrand and Jenks 2022). We also do not interpret low pH to be responsible for the low concentration of dsDNA in our stained samples. This is because the pH of buffered Lugol's iodine is neutral (Dawood et al. 2021), whereas a comparable DNA concentration drop was observed for buffered as well as unbuffered stains. Instead, our use of a dsDNA test kit may have been a factor. dsDNA Qubit assays require the DNA to be in a double-helical arrangement (Seshadri 2019). The low concentrations we report imply that dsDNA was not present in large quantities; therefore, we hypothesize that iodine contrast enhancement may accelerate the degradation of dsDNA into single-stranded DNA. If true, this would mean that single-stranded DNA sequences may remain intact—considering the buffered Lugol's fragmentation results above—but we are unable to quantify them using a dsDNA kit. We recommended that future studies quantify DNA concentrations using single- and double-stranded kits to test for this possibility.

### Molecular interactions of triiodide

We are aware of only two prior molecular analyses of post-diceCT specimen tissue (Green et al. 2017; Early et al. 2020). Using a PAXgene tissue fix solution (Qiagen, PreAnalytiX, Hombrechtikon Switzerland, cat # 765312) instead of formaldehyde fixation, Green et al. (2017) recovered ribonucleic acid (RNA) from embryonic, post-diceCT samples. This was in contrast to formaldehyde-preserved specimens, which yielded no meaningful RNA signal in their study (Green et al. 2017). Early et al. (2020), using proteomic and demineralization analyses, demonstrated that unbuffered diceCT stains generally reduced the amount of protein (from muscle and bone) and calcium (from bone) recovered from adult songbird specimens after staining. Amino acid modifications were also documented in this study, and they were interpreted to result from iodine covalently bonding with tyrosine and histidine (Early et al. 2020). What remains unclear is whether buffering the stain solution further mitigates the deleterious effects of triiodide on specimen tissues at the molecular level beyond what we have presented here. Until further data are available, we recommend that diceCT users proceed with both caution and the knowledge that even buffered Lugol's iodine stains likely permanently alter study specimens at the molecular level (see recommendations below).

### Future analyses

The results of this study are promising in that buffered Lugol's iodine preserves DNA integrity comparable to that of our control specimens and suggest that important next steps in DNA amplification and sequencing would produce similar quality sequences to those generated from formalin-preserved specimens. For example, aDNA protocols are tailored to maximize the recovery and sequencing success of degraded DNA, especially from samples that have been chemically treated (Straube et al. 2021). Designing primers or baits per aDNA methodologies accounts for the fragmented nature of the DNA, the potential chemical modifications, and the low concentration of endogenous DNA. To capture DNA sequences from preserved museum specimens, generally, and those previously subjected to a buffered Lugol's iodine solution, specifically, we recommend that researchers design primers or baits following aDNA protocols (e.g., Furtwängler et al. 2020; Kapp et al. 2021; Sundararaman et al. 2023). Previous research has demonstrated that the pH of preservation media does affect read quality and alignment (Hahn et al. 2022), with low pH causing depurination and oxidation. These ultimately result in a false, low guanine–cytosine content in sequences (Alqahtani et al. 2020). We predict, therefore, that specimens subjected to unbuffered diceCT stains will produce sequences with reduced fragment sizes and low guanine–cytosine content. Future research testing this hypothesis on controlled and museum specimens will be necessary to develop optimal protocols for staining alongside DNA extraction, amplification, and sequencing.

## Conclusion

Museum collections represent critical aspects of our biological heritage, and conserved approaches to destructive sampling requests are often reasonable. Herein, we test whether or not concerns over DNA loss following diceCT-based iodine staining are supported, focusing on new buffered iodine staining protocols. Our findings show that buffered Lugol's iodine preserves DNA fragment lengths but not concentrations when measured using dsDNA analyses. Future work should evaluate single-stranded kits for quantifying DNA concentrations after diceCT staining. Altogether, our findings implicate the following best practices to reconcile the use of the same tissues or specimens for 3D morphology and molecular sampling by the integrative organismal research community.

For curators, collections managers, and museum staff, we recommend the following:

1. If practical (e.g., if it does not disrupt anatomical regions of interest), extract samples for molecular analyses prior to contrast-enhanced imaging.
2. If (1) is not practical, then use buffered Lugol's iodine for iodine-based soft-tissue contrast enhancement.
3. Regardless of the method of contrast enhancement, notify future researchers sampling DNA about the specimen's staining history.
4. Recommend that genetic sampling of post-diceCT specimens follows phenol:chloroform:isoamyl extraction protocols.
5. Ensure that genetic sampling of post-diceCT specimens targets the heart whenever possible.
6. If specimens are numerous, and the project design does not involve sampling molecules and morphology from the same specimen(s), then allocating some specimens for 3D morphology and others for genomic documentation is also a reasonable approach.

For diceCT users, we recommend the following:

1. Preferentially employ buffered Lugol's iodine for iodine-based soft-tissue contrast enhancement.
2. For specimens stored in ethanol, seek prior approval to progress the samples through a series of hydration baths before staining as preparation for use of aqueous B-Lugol solutions.
3. If hydration steps were applied to samples (or specimens) prior to staining, and if the samples will ultimately be returned to ethanol for long-term museum storage after staining and study, step the samples through a series of dehydration baths to ensure sample equilibrium with the storage media.

With these best practices in mind, we support the viability of DNA sampling of post-diceCT specimens.

## Supplementary Material

obae017_Supplemental_File

## Data Availability

All data are available as tables within the main text and via Supplementary Tables S1 and S2.
